# Accurate Identification of *Ilex* (Aquifoliaceae) Taxa Based on Leaf Morphology Using Deep Learning

**DOI:** 10.3390/plants15091365

**Published:** 2026-04-29

**Authors:** Lin Yang, Yizhe Zhao, Cheng Jin, Shichang Wu, Zeyu Lu, Mingzhuo Hao, Changwei Bi, Kewang Xu

**Affiliations:** 1 State Key Laboratory of Tree Genetics and Breeding, Co-Innovation Center for Sustainable Forestry in Southern China, Key Laboratory of Tree Genetics and Biotechnology of Educational Department of China, College of Life Science, Nanjing Forestry University, Nanjing 210037, China; 8241811902@njfu.edu.cn (L.Y.);; 2 College of Information Science and Technology & Artificial Intelligence, Nanjing Forestry University, Nanjing 210037, China; jincheng@njfu.edu.cn (C.J.);

**Keywords:** *Ilex*, leaf classification, deep learning, convolutional neural network, Grad-CAM, taxa identification

## Abstract

Holly (*Ilex* L.) is a genus of woody dioecious plants with substantial ecological and economic value. However, its high species diversity and morphological similarity make accurate identification challenging. To address this, we constructed a multi-taxon *Ilex* leaf image dataset. We then trained six deep learning models—GoogLeNet, ResNet50, ResNet101, DenseNet121, DenseNet169, and EfficientNet-B3—using a unified PyTorch framework on cloud computing resources. Leaf images were preprocessed by background removal, resizing, cropping, and normalization. Model performance was evaluated using accuracy, F1-score, and Grad-CAM visualizations. Under an image-level data split that may overestimate generalization, all six models achieved over 99% classification accuracy on preprocessed leaf images under controlled laboratory conditions. DenseNet121 and DenseNet169 performed best, reaching 99.65% accuracy. Because images of the same leaf or same plant could appear in both training and test sets under this split, plant-level cross-validation is required to assess real-world generalizability. The reported accuracies represent an upper-bound estimate under image-level splitting. The framework offers a rapid and accurate tool for preliminary screening under controlled conditions, but its performance on raw field photographs and across different collection sites remains to be validated.

## 1. Introduction

*Ilex* L., belonging to the family Aquifoliaceae, represents a key component of global greening efforts and ecosystem functioning. It is also the largest extant genus of woody dioecious plants, comprising more than 600 species worldwide [1]. The genus has a broadly pantropical to subtropical distribution, but species richness is highly uneven across regions. East Asia, as well as Central and South America, each harbor more than 200 *Ilex* species. Southeast and South Asia contain approximately 120 species. In contrast, North America supports only 20–30 species. Fewer than five species occur in each of sub-Saharan Africa, Australia, Europe, and the islands of the Pacific and Atlantic Oceans [2,3]. In addition to its ecological significance, *Ilex* has substantial economic value. Many species are widely cultivated as ornamental plants, including *Ilex opaca* Aiton (American holly), *I. cornuta* Lindl. & Paxton (Chinese holly), and *I. aquifolium* L. (English holly) [4,5]. Moreover, several species are used for beverage production, such as *I. kaushue* S.Y. Hu and *I. latifolia* Thunb. (Kuding tea) [6,7], as well as *I. paraguariensis* A.St.-Hil. [8,9], the source of yerba mate.

Like many species-rich genera, the infrageneric classification of *Ilex* has long been challenging and has undergone repeated revisions since Linnaeus established the genus [10]. Different taxonomists, including Gray [11], Loesener [12,13,14,15], Hu [16], Maguire [17], and Galle, have proposed morphology-based classification systems. These systems mainly rely on reproductive organs such as inflorescences, flowers, and fruits. Leaf traits—including shape, apex, margin, and venation—are only auxiliary indicators. Vegetative organs like leaves exist throughout the growth cycle. Therefore, they are more convenient and stable for species identification in practical applications. Leaf traits remain fundamental diagnostic characters. They are essential for germplasm collection, varietal trait characterization, and genetic resource conservation and utilization [18,19]. However, traditional manual identification is time-consuming. It is highly dependent on expert experience, especially under large-scale, high-diversity conditions. This makes it difficult to achieve both efficiency and accuracy [20,21]. Even computer-assisted approaches based on conventional single-machine image classification face growing limitations in memory, storage, processing speed, and data throughput. These restrictions limit their applicability in modern big-data environments [22]. In vegetable crops, artificial intelligence has been widely adopted for phenotypic data acquisition, multi-omics analysis, and intelligent production [23]. For example, multispectral imaging combined with partial least squares discriminant analysis has been used to accurately identify subtle leaf color differences in Chinese cabbage [24]. Furthermore, phylogenomic studies have revealed widespread incomplete lineage sorting and hybridization within *Ilex*. Tools such as Phytop [25] have been developed to visualize and quantify these signals from species trees. With the rapid development of deep learning, image recognition techniques have shown remarkable potential in plant species classification [26,27,28]. Therefore, constructing deep learning-based leaf image classification models for *Ilex* offers a promising way to improve classification efficiency and accuracy. This has important implications for ecological monitoring, species conservation, horticultural breeding, and biodiversity research.

Convolutional neural networks (CNNs) have become a cornerstone of modern computer vision, particularly for image classification tasks. Unlike traditional machine learning methods that rely on handcrafted features, CNNs learn hierarchical representations directly from raw pixel data. Lower layers capture edges and textures. Deeper layers combine these into semantic concepts such as leaf shapes, margins, and venation patterns. This ability to automatically learn discriminative features makes CNNs well suited for fine-grained plant identification, where subtle morphological differences distinguish closely related taxa. In recent years, deep learning has achieved notable success in agricultural and horticultural image classification [29,30]. For example, Liu et al. [31] developed a deep learning method for large-flowered chrysanthemum cultivar recognition, achieving 78% accuracy on images collected from different years. In addition, Zhang et al. [32] designed a 13-layer CNN for fruit category classification in factories and supermarkets, reporting an overall accuracy of 94.94%. In a study investigating olive cultivars, Ponce et al. [33] applied the Inception-ResNetV2 architecture to olive cultivar identification, obtaining 95.91% precision; Sennan [34] optimized residual block connections to enhance fine-grained feature extraction for spinach cultivar classification; Zhu et al. [35] applied an EfficientNet-B4 model with attention mechanism to identify oil tea cultivars; Osako [36] constructed a litchi cultivar classification model based on VGG-16, reaching 98.33% accuracy through morphology-adaptive preprocessing and parameter tuning; and Jung [37] developed a deep learning-based detection model that integrates multidimensional image features for effective identification of pine wilt disease. Despite these advances, deep learning applications specifically for *Ilex* classification remain scarce. Existing studies typically include only a small number of common taxa, which fails to capture the extensive species diversity of the genus and therefore limits model generalizability. Furthermore, most existing models are trained on images with idealized backgrounds and do not incorporate effective strategies for handling complex field conditions, such as cluttered backgrounds, variable illumination, or partial occlusion. As a result, fine-grained discrimination among closely related *Ilex* taxa remains insufficient to meet the practical demands of germplasm surveys, ecological monitoring, and horticultural breeding.

To address these limitations, we first constructed a self-built *Ilex* leaf image dataset focusing specifically on leaf morphology. It includes representative taxa such as *I. cornuta*, *I. latifolia*, *I. rotunda* Thunb, and *I. pubescens* Hook. & Arn. We incorporated leaf samples from multiple individuals per taxon to enhance data diversity and improve model robustness. Based on this dataset, we conducted model training within the PyTorch framework using cloud-computing resources. This ensured sufficient computational efficiency and scalability. We trained six CNN architectures—GoogLeNet, ResNet50, ResNet101, DenseNet121, DenseNet169, and EfficientNet-B3—to develop *Ilex* taxa classification models. By combining a targeted dataset with cloud-based training and parallel evaluation of multiple representative CNN architectures under consistent conditions, this study enables an objective assessment of model performance and suitability for *Ilex* leaf classification.

The objectives of this study are as follows:

(1) To construct a high-quality, multi-taxon and laboratory-based *Ilex* leaf image dataset to support robust model training;
(2) To train and evaluate *Ilex* taxa classification models based on leaf morphological features using six CNN architectures within the PyTorch framework and cloud-based training environment;
(3) To systematically compare the performance of these CNN models—including accuracy, recall, and parameter efficiency—and to identify efficient architectures best suited for *Ilex* leaf morphology, thereby providing a technical foundation for germplasm resource surveys and practical taxa identification applications within the genus *Ilex*.

## 2. Materials and Methods

### 2.1. Image Acquisition

The RGB image dataset of *Ilex* used in this study was collected in Jiangning and Xuanwu Districts, Nanjing, Jiangsu Province. Sampling sites included the Jiangsu Academy of Forestry Sciences (latitude: 31.86° N, longitude: 118.78° E) and the *Ilex* base of Nanjing Forestry University (latitude: 32.22° N, longitude: 118.83° E). The region has an average annual high temperature of 23 °C, an average annual low temperature of 14 °C, and a mean annual temperature of 17 °C. It has sufficient precipitation, providing a suitable climate for *Ilex* growth. Leaves were photographed from 3 to 5 mature individual plants per taxon, except for rare cultivars where only 2 plants were available. From each plant, we collected 20–60 fully expanded leaves, covering both adaxial and abaxial surfaces. The exact number varied depending on plant size and leaf availability. The developmental stage was uniformly mature leaves—fully expanded and without senescence. The camera-to-leaf distance was approximately 30–50 cm. We adjusted it slightly to keep the leaf occupying about 70% of the frame. Sampling was conducted from May to August 2025. The photography equipment was a Nikon D7500 digital camera (Nikon Corporation, Tokyo, Japan) equipped with a Micro 105 mm lens (Nikon Corporation, Tokyo, Japan). Images were captured daily between 09:00 and 17:00. A total of 45 *Ilex* taxa were photographed, including varieties, cultivars, and hybrids. We took 200–400 images per taxon in total, including both adaxial and abaxial leaf surfaces. This resulted in 11,500 images overall, including *Ilex latifolia*, *I. cornuta*, *I. rotunda*, *I. pernyi* Franch, and other taxa (Table S1). All samples were collected from the *Ilex* germplasm collection of Nanjing Forestry University, where each taxon is maintained as living plants with documented accession records and verified taxonomic names (following Flora of China [18]). No voucher specimens were collected due to conservation restrictions, but the living reference plants are permanently maintained in the germplasm collection and can be accessed for verification. The image dataset is available from the corresponding author upon reasonable request.

### 2.2. Convolutional Neural Network Models

Since AlexNet achieved breakthrough performance on the ImageNet dataset [38], CNNs have become central to computer vision research. Subsequent studies, including the VGG network, demonstrated that increasing network depth can significantly improve classification accuracy [39]. However, the use of nonlinear activation functions such as ReLU introduces irreversible information loss. This loss may accumulate with increasing depth and lead to vanishing gradient problems during training. To overcome these limitations, ResNet introduced residual connections. These allow information to bypass intermediate layers, enabling effective training of deeper networks and improving performance [40]. Building upon this idea, DenseNet employs dense connectivity to promote efficient feature reuse and stable gradient propagation. This offers advantages in small-sample and fine-grained classification tasks, where a balance between accuracy and model complexity is required. All six models were initialized with weights pre-trained on the ImageNet1K dataset. The final fully connected layer was replaced with a new layer matching the number of *Ilex* classes (45). The overall experimental configuration is detailed in Table 1. During the first 5 epochs, only the newly added classification layer was trained with a learning rate of 0.0003 (Table 2, Phase I LR), while the backbone remained frozen. For the subsequent 25 epochs, the entire network was fine-tuned with a learning rate of 0.00003 (Table 2, Phase II LR). This transfer learning strategy accelerated convergence and improved generalization.

#### 2.2.1. GoogLeNet Model

GoogLeNet is a deep convolutional neural network architecture proposed by Google in 2014 and achieved first place in the classification task of the ImageNet Large Scale Visual Recognition Challenge in the same year [41]. The primary innovation of GoogLeNet lies in the introduction of the Inception module, which captures multi-scale features by employing convolutional kernels of different sizes, thereby reducing the number of parameters while improving computational efficiency. As the core component of GoogLeNet, the Inception module is designed to achieve multi-scale perception by processing input features through parallel convolutional operations and subsequently fusing the extracted features. Its basic structure consists of four parallel branches: 1 × 1 convolution, 3 × 3 convolution, 5 × 5 convolution, and 3 × 3 max pooling. Furthermore, the incorporation of 1 × 1 convolutions prior to larger convolutional kernels effectively reduces dimensionality, thereby decreasing both parameter count and computational cost.

#### 2.2.2. ResNet50 Model

ResNet50 is an important member of the residual network (ResNet) family, in which residual learning and identity shortcut connections effectively mitigate the vanishing gradient problem in deep neural networks, thereby enabling stable training of a 50-layer convolutional architecture. The fundamental principle of ResNet is to learn a residual function F(x) = H(x) − x instead of directly approximating the underlying mapping H(x), allowing gradients to propagate efficiently through shortcut paths during backpropagation. The ResNet50 architecture consists of three main components. The head includes a 7 × 7 convolutional layer with a stride of 2, followed by a 3 × 3 max-pooling layer with a stride of 2, which performs rapid spatial downsampling and extracts initial feature representations. The body is composed of four groups of bottleneck residual blocks with output channel dimensions of 256, 512, 1024, and 2048, respectively; in each group, the first block performs downsampling with a stride of 2, while the remaining blocks maintain spatial resolution. The tail comprises a global average pooling layer followed by a fully connected layer for classification.

#### 2.2.3. ResNet101 Model

ResNet101 is a deep residual network that mitigates the degradation problem in very deep neural networks through residual learning. The architecture comprises 101 layers and is built from multiple bottleneck residual blocks, beginning with a 7 × 7 convolutional layer followed by a 3 × 3 max-pooling layer, and subsequently four convolutional stages (conv2_x–conv5_x) containing stacked residual blocks. Each bottleneck block consists of a sequence of 1 × 1, 3 × 3, and 1 × 1 convolutional layers, combined with batch normalization and ReLU activation, enabling efficient feature transformation and stable gradient propagation. The network ends with a global average pooling layer and a fully connected layer, with classification outputs produced via a softmax function.

#### 2.2.4. DenseNet121 Model

DenseNet121 is a convolutional neural network that improves information flow and gradient propagation through dense connectivity, in which each layer receives feature maps from all preceding layers [42]. This connectivity pattern effectively alleviates the vanishing gradient problem, enhances feature reuse, and reduces the number of model parameters. The architecture consists of multiple dense blocks and transition layers. Each dense block comprises a series of convolutional layers, including 1 × 1 bottleneck convolutions followed by 3 × 3 convolutions, whose outputs are concatenated and used as input for subsequent layers. Transition layers include 1 × 1 convolutions and 2 × 2 average pooling operations to perform feature map downsampling. By enabling direct access to previously learned features, DenseNet121 allows for a narrower network design with fewer channels per layer, while the dense connections also provide an implicit regularization effect that helps reduce overfitting.

#### 2.2.5. DenseNet169 Model

DenseNet169 is a deep convolutional neural network designed for image classification, in which dense connectivity and feature reuse are employed to improve model performance while reducing parameter count and computational cost. The architecture is composed of dense blocks and transition layers. Within each dense block, every layer is directly connected to all preceding layers, which accelerates information flow, mitigates the vanishing gradient problem, and enhances feature sharing across the network. Transition layers are used to control feature map dimensions and reduce computational complexity. Compared with residual networks, DenseNet169 typically requires fewer parameters while maintaining competitive or superior classification performance, and its channel-wise feature concatenation enables effective feature reuse, making it particularly well suited for small-sample datasets.

#### 2.2.6. EfficientNet-B3 Model

EfficientNet is a convolutional neural network architecture optimized using a compound scaling strategy that jointly scales network depth, width, and input resolution to achieve improved performance and efficiency [43]. EfficientNet-B3 is a representative variant in this family with a specific parameter configuration. Its architecture is composed of multiple stages, beginning with a 3 × 3 convolutional layer with a stride of 2, followed by batch normalization and Swish activation, and subsequently several stages formed by stacked Mobile Inverted Bottleneck Convolution (MBConv) modules. The network ends with a 1 × 1 convolutional layer with batch normalization and Swish activation, followed by global average pooling and a fully connected classification layer. The MBConv module, which serves as the core building block of EfficientNet, consists of an expansion 1 × 1 convolution, a k × k depthwise convolution (k = 3 or 5), a squeeze-and-excitation (SE) module, and a projection 1 × 1 convolution with linear activation, with dropout applied to improve regularization. This design enables EfficientNet-B3 to achieve a favorable balance between classification accuracy and computational cost.

**Figure 1:**
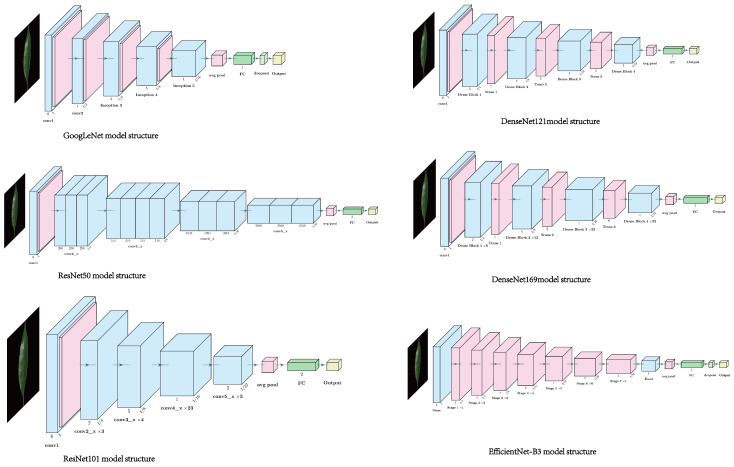
Six model structure diagrams are shown from left to right: GoogLeNet, ResNet50, ResNet101, DenseNet121, DenseNet169, and EfficientNet-B3.

Several mainstream deep convolutional neural network (CNN) architectures have been widely applied to image classification tasks, including VGG, GoogLeNet, and ResNet, which represent state-of-the-art large-scale network designs. To systematically compare the characteristics and advantages of different CNN architectures when applied to *Ilex* leaf image classification, this study selected six representative CNN models—GoogLeNet, ResNet50, ResNet101, DenseNet121, DenseNet169, and EfficientNet-B3—for model training and evaluation on the self-constructed *Ilex* leaf dataset (Figure 1). These models were chosen because they differ substantially in network depth, connectivity patterns, and parameter efficiency, allowing a comprehensive assessment of their adaptability and performance in fine-grained *Ilex* taxa classification tasks.

## 3. Experiment

### 3.1. Hardware and Software Environment

All experiments were conducted on the Featurize cloud computing platform. It was equipped with an NVIDIA RTX 4090 GPU (24 GB video memory) (NVIDIA Corporation, Santa Clara, CA, USA), an AMD EPYC 9354 CPU (16 cores) (AMD, Inc., Santa Clara, CA, USA), 56 GB of RAM, and 700 GB of storage. The experimental environment was deployed using Docker (v20.10.10). Python (v3.10.12) was used as the programming language. PyTorch (v2.0.1) served as the deep learning framework. Detailed specifications are provided in Table 1. The dataset was split at the image level using stratified random sampling into 70% training, 15% validation, and 15% test sets. The validation set was used only for early stopping and model checkpoint selection. The test set was held out for final unbiased evaluation and was never used during training or any model selection decision. We acknowledge that this image-level split may result in different images of the same leaf or the same individual plant appearing in multiple sets. This could lead to an optimistic estimate of generalization performance. Therefore, the reported accuracies (all based on the test set) should be interpreted as an upper-bound benchmark under ideal split conditions. The dataset was organized using a class-based directory structure, in which each *Ilex* taxon corresponded to a separate subfolder. All conclusions about model generalization in this study are limited to this image-level split.

### 3.2. Dataset Preprocessing

The collected images were subjected to preprocessing prior to model training. The rembg tool (version 2.0.50, commit a9c2a39) was employed to remove complex backgrounds, and images with blurring or poor visual quality were identified and discarded to ensure dataset reliability [44]. All images were resized to a uniform resolution and subsequently standardized and normalized. In addition, data augmentation techniques were applied to enhance sample diversity and reduce the risk of model overfitting.

#### 3.2.1. Image Resizing

Image resizing ensures that all input images share a consistent scale, a fundamental requirement for many deep learning models. This process also reduces computational complexity and accelerates model training efficiency. The input resolution for all models was set to 224 × 224 pixels. The specific preprocessing steps to achieve this resolution differed between the training set and the validation/test sets, as detailed in Section 3.2.3. Although EfficientNet-B3 was originally designed for larger inputs such as 300 × 300 pixels, we used the common resolution of 224 × 224 across all six models to ensure a fair comparison of architectural differences. EfficientNet-B3 can accept 224 × 224 inputs, and using a unified resolution avoids introducing confounding factors related to input size.

#### 3.2.2. Normalization

Normalization scales image pixel values to a specified range ([0, 1]), ensuring consistency across input data and facilitating more stable model training. This process reduces the influence of illumination variations and makes feature learning more effective. In addition, normalization aims to minimize bias and errors caused by differences in feature scales, thereby improving the robustness and convergence behavior of deep learning models.

#### 3.2.3. Data Augmentation

To improve model robustness and generalization, online data augmentation was applied exclusively to the training set. Each training image was randomly transformed using the following sequence: a random resized crop to 224 × 224 pixels with a scale range of 0.8 to 1.0 and an aspect ratio range of 3/4 to 4/3, a random horizontal flip with a probability of 0.5, a random rotation within ±20 degrees, a random translation within ±10% of the image size, and a color jitter adjusting brightness, contrast, and saturation by a factor uniformly sampled from 0.7 to 1.3. The validation and test sets were not augmented; they were only resized to 256 × 256 pixels, center-cropped to 224 × 224 pixels, converted to a tensor, and normalized with the same statistics. All transformations were implemented using PyTorch’s torchvision.transforms. No augmentation increased the stored dataset size.

### 3.3. Training Configuration

#### 3.3.1. Hyperparameters

All models were trained and evaluated on the holly leaf dataset using a unified set of hyperparameters to ensure fair comparison. The detailed hyperparameter configurations are presented in Table 2.

#### 3.3.2. Loss Function

We adopted the cross-entropy loss function for model training and evaluation. CrossEntropyLoss is one of the most widely used loss functions for multi-class classification tasks. It operates on an n-dimensional input. The input is first processed by the Softmax function to transform raw outputs into a probability distribution over all class labels. These probabilities are then converted into log probabilities. The negative log-likelihood function computes the loss by comparing the predicted log probabilities with the true class labels [45]. This mechanism penalizes incorrect predictions and encourages the model to assign higher probabilities to the correct classes.

Specifically, CrossEntropyLoss optimizes model performance by maximizing the probability of the correct class while minimizing the probabilities of incorrect classes. This loss function is particularly suitable for multi-class classification problems. It explicitly models the probabilistic relationships among multiple categories and ensures sensitivity to class-specific prediction errors. As training progresses, a decreasing cross-entropy loss indicates improved classification capability. The mathematical formulation is shown in Equation (1), where *yi* represents the one-hot encoded ground-truth label and pi denotes the predicted probability of class i.

(1) $$Loss=-\sum_{i=1}^{N}y_{i}log(p_{i})$$

#### 3.3.3. Optimizer

We chose Adam as the optimizer. The learning rate was scheduled using ReduceLROnPlateau (Table 2): whenever the validation loss plateaued for three consecutive epochs, the learning rate was multiplied by 0.5. During the first five epochs, only the newly added classification layer was trained with a fixed learning rate of 0.0003. After unfreezing all layers at epoch five, the learning rate was reset to 0.00003, and the ReduceLROnPlateau scheduler continued to monitor the validation loss for the remaining 25 epochs.

To further improve training stability and generalization, we applied label smoothing (ε = 0.1), mixup augmentation (α = 0.2, applied only after unfreezing), weighted random sampling to balance class frequencies, and gradient clipping (max norm = 1.0).

#### 3.3.4. Reproducibility and Uncertainty

All experiments used a fixed random seed of 42 for data splitting, initialization, and augmentation. For each model, we derived a unique seed from the base seed. This ensured that different models had independent random states while maintaining overall reproducibility. The final model checkpoint was selected based on the highest validation accuracy during training, using early stopping with a patience of 10 epochs. The source code that supports the findings of this study has been deposited in a public GitHub repository: https://github.com/chengJ914/Accurate-Identification-of-Ilex-Aquifoliaceae-Taxa-Based-on-Leaf-Morphology (accessed on 24 April 2026). The five-fold cross-validation fold assignments (folds.csv) are provided as Supplementary Materials. The dataset is available from the corresponding author upon reasonable request due to germplasm conservation restrictions.

### 3.4. Evaluation Metrics

To comprehensively evaluate the effectiveness of the proposed models, we employed a set of performance metrics. These metrics provide a quantitative assessment of predictive accuracy and offer insights into the strengths and limitations of the models when handling complex data. The following subsections describe the definition, significance, and calculation of each metric. All metrics were calculated based on true positives (TP), true negatives (TN), false positives (FP), and false negatives (FN). In addition, confusion matrices were used to assess classification performance.

For multi-class classification tasks, the F1-score was calculated by first computing precision and recall for each class and then averaging them across all classes [46]. The F1-score is the harmonic mean of precision and recall. It reflects the model’s overall classification performance across different categories. A higher F1-score indicates that the model not only achieves accurate predictions but also identifies most positive samples. Accuracy represents the proportion of correctly classified samples relative to the total number of samples. Precision measures the proportion of true positive samples among all samples predicted as positive. Recall indicates the proportion of actual positive samples that are correctly identified. The mathematical definitions are provided in Equations (2)–(5).

(2) Accuracy = (TP + TN)/(TP + TN + FP + FN) × 100%

(3) Recall = TP/(TP + FN) × 100%

(4) Precision = TP/(TP + FP) × 100%

(5) F1-score = (2 × Precision × Recall)/(Precision + Recall) × 100%

For multi-class classification, the reported precision, recall, and F1-scores are macro-averaged—calculated independently for each class and then averaged without weighting by class support. Macro-averaging treats all taxa equally, regardless of sample size, which is appropriate for evaluating performance across the full taxonomic diversity of the dataset. Because the dataset has a balanced class distribution (200–400 images per taxon), micro-averaged values yield nearly identical results.

### 3.5. Five-Fold Cross-Validation

To further assess the robustness of the model, we performed five-fold cross-validation on EfficientNet-B3. The entire dataset was partitioned into five approximately equal-sized folds using stratified random sampling based on class labels, with a fixed random seed of 42 to ensure reproducibility. For each fold, the model was trained on the combination of the other four folds and evaluated on the held-out fold. This procedure was repeated five times, with each fold serving as the test set once. The mean accuracy and standard deviation across the five folds were calculated. We acknowledge that this cross-validation was performed at the image level, meaning that different images of the same leaf or the same individual plant could appear across different folds. Therefore, these results represent an assessment of model stability under random image-level partitions only, not plant-level generalization. Plant-level cross-validation, where all images from a single plant are assigned exclusively to one partition, will be required for future field-ready evaluations. The exact fold assignment for each image (i.e., which fold each image belongs to) is provided in Supplementary File folds.csv.

For the five-fold cross-validation, we used a simplified training configuration (fixed learning rate of 0.0003, CosineAnnealing scheduler, input size 300 × 300, and no label smoothing or mixup) to reduce computational cost. The main conclusions about model stability remain valid.

## 4. Results

### 4.1. Comparison of Classification Performance Among CNN Models

To evaluate performance, we selected six representative CNN models: GoogLeNet, ResNet50, ResNet101, DenseNet121, DenseNet169, and EfficientNet-B3. These models were chosen for their proven effectiveness in visual recognition tasks and their diverse architectural designs. This allowed a comprehensive assessment under identical experimental conditions.

As shown in Table 3, all six models achieved excellent classification performance on the *Ilex* dataset, with accuracies exceeding 99%. GoogLeNet achieved 99.59% accuracy. The ResNet series performed well, with ResNet50 reaching 99.59% accuracy. ResNet101 showed slightly lower accuracy (99.48%) compared to ResNet50. This may be attributed to its deeper network and increased parameter complexity. The additional capacity could lead to a certain degree of overfitting, preventing further improvement for this dataset.

For DenseNet and EfficientNet architectures, DenseNet121 achieved 99.65% accuracy, and DenseNet169 also reached 99.65%, both being the highest among all models. EfficientNet-B3 obtained 99.19% accuracy. These results indicate that recent architectures emphasizing feature reuse and efficient scaling demonstrate strong performance in *Ilex* leaf classification under controlled laboratory conditions, although EfficientNet-B3 underperformed relative to the DenseNet variants in this specific dataset.

### 4.2. Model Complexity and Computational Efficiency

Regarding model complexity in terms of number of parameters, GoogLeNet had 6.6 million parameters, DenseNet121 had 8.0 million, EfficientNet-B3 had 12.0 million, DenseNet169 had 14.2 million, ResNet50 had 25.6 million, and ResNet101 had 44.5 million. Notably, ResNet101 has more parameters than ResNet50 but achieved slightly lower accuracy, from 99.59 percent to 99.48 percent, suggesting that increased parameter count does not guarantee better classification performance on this dataset. Architectural design and feature utilization appear more critical than parameter quantity alone.

### 4.3. Training and Validation Curve Analysis

**Figure 2:**
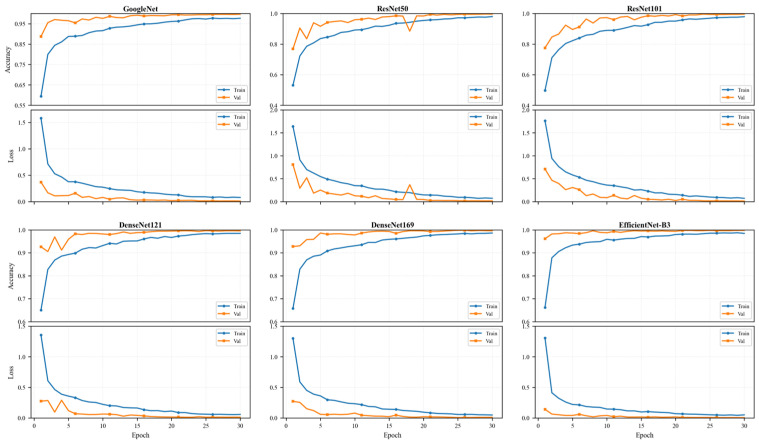
Training and validation of loss–accuracy curves of six CNN models. Blue indicates training, and yellow indicates validation.

Figure 2 illustrates the training and validation accuracy and loss curves of six deep learning models, namely GoogLeNet, ResNet-50, ResNet-101, DenseNet-121, DenseNet-169, and EfficientNet-B3. For all models, training and validation accuracies show a consistent upward trend with increasing epochs, while the corresponding loss values decrease rapidly in the early training stage and gradually stabilize thereafter. This behavior indicates effective model learning and good convergence.

Notably, the validation curves follow similar trends to the training curves across all architectures, suggesting strong generalization performance without evident overfitting. Most models reach high validation accuracy (>0.95) within the first five to ten epochs, reflecting fast convergence and efficient optimization. Among them, EfficientNet-B3 and DenseNet variants exhibit particularly smooth and stable convergence behavior, achieving the highest accuracy with very low loss values. The final validation accuracies exceed 99% for all models, consistent with the test set results reported in Table 3.

### 4.4. Confusion Matrix Analysis

**Figure 3:**
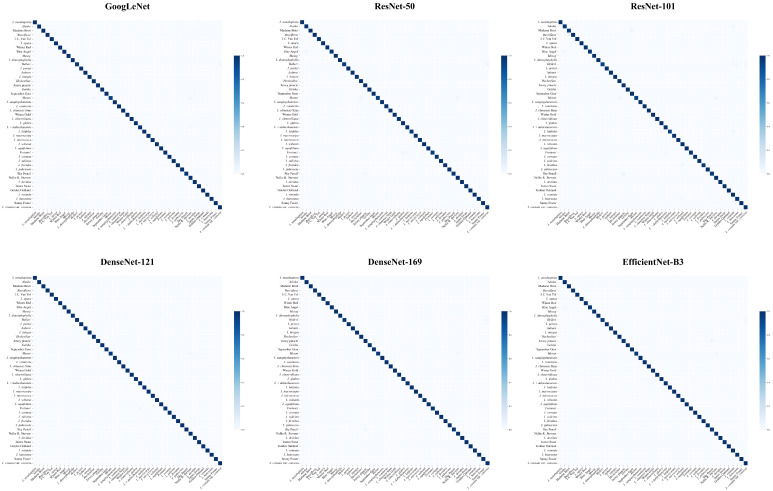
Confusion matrices of six CNN models. Diagonal cells indicate correct classifications, and off-diagonal cells indicate misclassifications.

Figure 3 presents the confusion matrices of GoogLeNet, ResNet50, ResNet101, DenseNet121, DenseNet169, and EfficientNet-B3 evaluated on the test set. Overall, the prediction results of all models are mainly concentrated along the diagonal of the confusion matrices, indicating that most samples were correctly classified and that the models achieved good overall classification performance.

For all network architectures, the off-diagonal regions exhibit relatively low intensity, with only sporadic misclassifications observed among a limited number of classes, suggesting strong discriminative capability between different categories. However, a closer examination reveals that almost all models exhibit relatively lower classification performance for *Ilex crenata* ‘Helleri’ compared to the macro-average, and it is frequently misclassified as *I*. *crenata* var. *convexa* Makino. In addition, *I.* × *meserveae* ‘Mesan’ is commonly confused with *I.* × *meserveae* ‘HeckenStar’ and *I.* × ‘Nellie R. Stevens’. Moreover, mutual misclassification is also observed between *I. crenata* ‘Jersey Pinnacle’ and *I. crenata* ‘Sky Pencil’.

Although the overall distribution patterns of the confusion matrices are similar across models, these class-specific misclassification patterns indicate subtle differences in feature extraction and class discrimination abilities among the different network architectures. Nevertheless, all models demonstrate stable and consistent overall classification performance.

### 4.5. F1-Score Distribution Across Taxa

**Figure 4:**
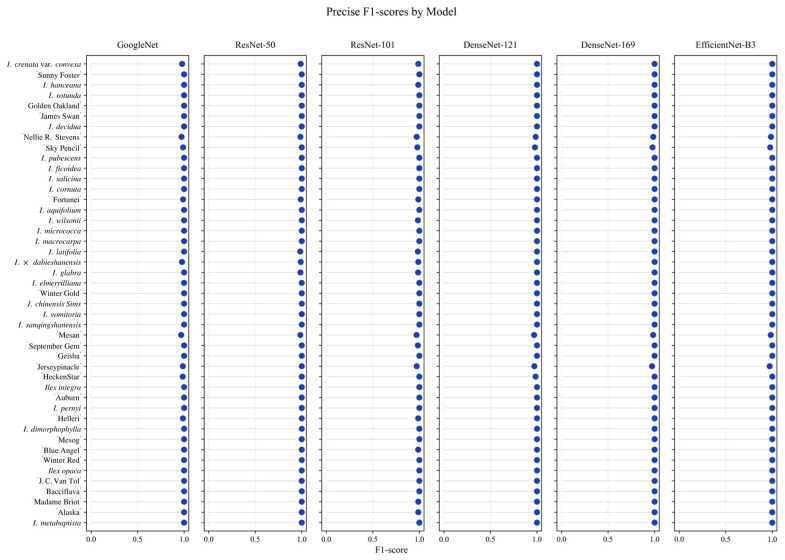
Macro-averaged F1-scores of six CNN models. Higher values indicate better classification performance.

Figure 4 illustrates the taxon-level F1-scores obtained by GoogLeNet, ResNet50, ResNet101, DenseNet121, DenseNet169, and EfficientNet-B3 on the test set. Overall, most *Ilex* taxa achieved consistently high F1-scores across all models, with values clustering close to 1.0, indicating robust and balanced classification performance at the taxa level.

For the majority of taxa, F1-scores showed minimal variation among different network architectures, suggesting that these taxa could be reliably distinguished regardless of model choice. This consistency implies that discriminative morphological features for these taxa were effectively captured by all evaluated models.

Slight reductions or fluctuations in F1-scores were observed for a limited number of taxa; however, these variations remained relatively small and did not substantially affect overall classification performance. Such minor differences may be attributable to interspecific similarity or subtle morphological variation among closely related taxa. In general, the uniformly high F1-scores across taxa demonstrate that the proposed deep learning models provide stable and reliable performance for fine-grained identification of *Ilex* taxa.

### 4.6. Grad-CAM Visualization and Feature Attention Analysis

Grad-CAM visualizations indicate that all *Ilex* leaf images were primarily attended to in key morphological regions, including the contour, margin, apex, and venation patterns, while background information was largely ignored [47]. This focused attention explains the misclassifications observed in the confusion matrices, particularly among morphologically similar taxa, such as closely related *I. crenata* cultivars.

**Figure 5:**
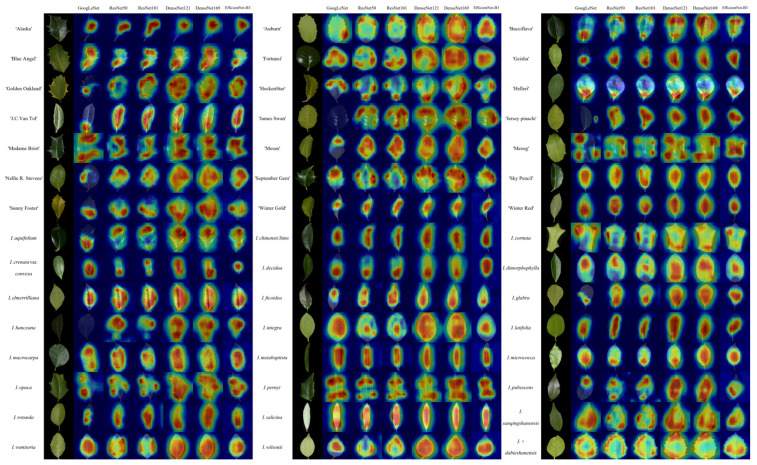
Grad-CAM heatmaps of six CNN models applied to *Ilex* leaf images. The leftmost column shows the original leaf images, followed from left to right by heatmaps of GoogLeNet, ResNet50, ResNet101, DenseNet121, DenseNet169, and EfficientNet-B3.

Figure 5 shows the original leaf images (left) and the corresponding attention heatmaps from six CNN models (right). Red and yellow regions indicate high attention, while blue regions indicate low attention. Attention patterns varied slightly across samples, reflecting model sensitivity to specific traits: spiny-margined leaves received more focus on the margin and apex, whereas elongated leaves emphasized the mid-lamina regions.

ResNet, DenseNet, and EfficientNet produced more continuous and comprehensive attention maps than GoogLeNet, effectively covering major morphological regions. These differences align with model architectures: ResNet’s residual connections preserve multi-scale features, DenseNet’s dense connections enable feature reuse, and EfficientNet’s compound scaling allows adaptive feature representation. Overall, the visualizations support the high F1-scores and highlight the models’ ability to capture discriminative leaf traits.

### 4.7. AI Holly Website

**Figure 6:**
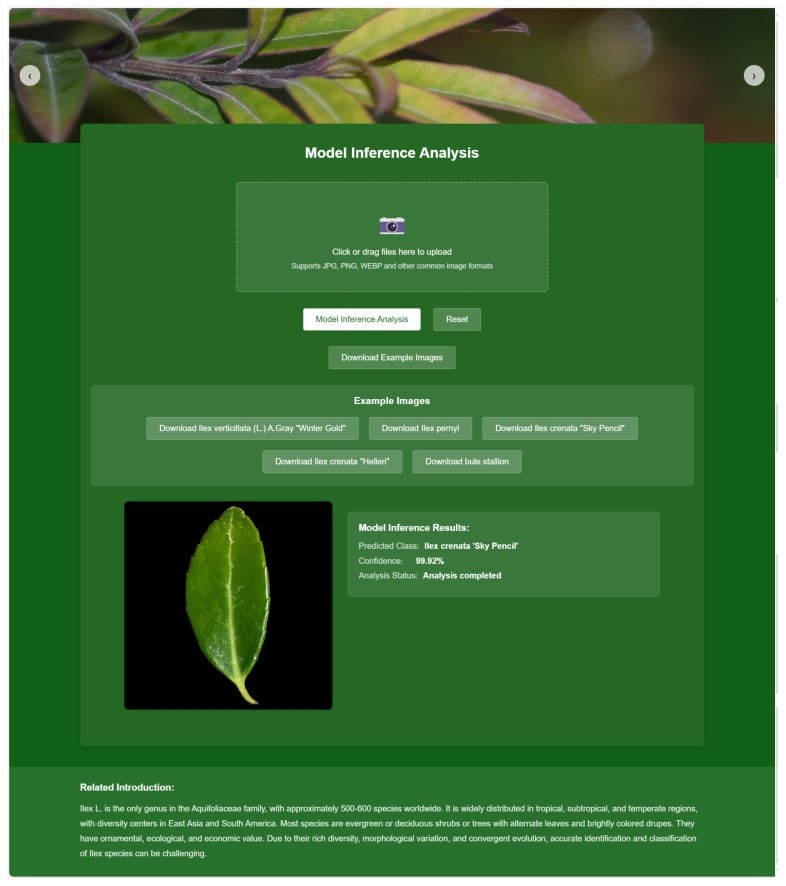
The interface of the AI Holly website.

To facilitate the use of our holly leaf classification model by researchers, we developed the AI Holly website (http://111.229.66.153/ (accessed on 20 March 2026)) (Figure 6). The platform deploys the DenseNet169 model and provides a streamlined, intuitive interface, allowing users to easily classify holly leaf images. Users only need to upload RGB leaf images in PNG or JPG format. Once uploaded, the images are processed by the classification model hosted on the server, which quickly returns the top three predicted taxa along with their respective probabilities, providing an efficient tool for researchers. Additionally, the website features a bar chart displaying the predicted accuracy for different taxa. No uploaded images or predictions are stored on the server.

On the backend, the uploaded images are first transferred to the server’s hard drive for temporary processing and then read by a Python script for a series of preprocessing operations. The images are resized to 224 × 224 pixels and then normalized using the mean and standard deviation derived from the ImageNet1K dataset. Before being input into the model, a new dimension is inserted into the 0th dimension of the three-dimensional image tensor, transforming it into a four-dimensional tensor. This step ensures compatibility with deep learning models that require a batch dimension for simultaneous processing of multiple images. Finally, using the trained model parameters and network architecture, the processed images are identified, enabling accurate classification of holly leaf taxa. This comprehensive preprocessing and recognition pipeline ensures that the input images are properly formatted and normalized, which is essential for achieving optimal model performance.

### 4.8. Five-Fold Cross-Validation Results

The five-fold cross-validation on EfficientNet-B3 yielded a mean test accuracy of 99.40% with a standard deviation of 0.12%. The accuracies for the five folds were 99.52%, 99.17%, 99.48%, 99.43%, and 99.39%, respectively. These results are consistent with the accuracy (99.19%) obtained from the single 70/15/15 split reported in Table 3 for EfficientNet-B3, indicating that the model’s performance is stable under the current image-level split. The low standard deviation across folds further indicates that the model’s performance is stable across different image-level random partitions. It is important to note that this five-fold cross-validation was also performed at the image level; therefore, it does not address the potential overestimation caused by having images of the same leaf or the same plant across different folds. Plant-level cross-validation is still required to estimate real-world generalizability.

## 5. Discussion

### 5.1. Overall Performance of CNN Architectures

This study demonstrates that modern CNN architectures can achieve highly accurate and stable classification of *Ilex* leaf taxa under controlled laboratory conditions using preprocessed images. All six models exceeded 99 percent accuracy, with F1-scores near 1.0 for most of the 45 taxa, indicating robust fine-grained performance. These results confirm that *Ilex* leaves possess highly discriminative morphological features that can be effectively captured by deep learning models. Among the six models, DenseNet121 and DenseNet169 achieved the highest overall accuracy of 99.65 percent, while GoogLeNet achieved 99.59% accuracy with fewer parameters (6.6 M). The ResNet series, including ResNet50 and ResNet101, also performed well, with ResNet50 reaching 99.59 percent accuracy.

### 5.2. Model Complexity and Generalization

Subtle performance differences were observed among architectures. ResNet and DenseNet variants outperformed GoogLeNet in accuracy and convergence, likely due to residual and dense connections enabling multi-scale feature preservation and reuse. EfficientNet-B3 further enhanced feature representation across leaf sizes via compound scaling. However, deeper networks such as ResNet101 with 44 million parameters did not consistently outperform shallower ones like ResNet50 with 25 million parameters. This can be attributed to the mismatch between dataset characteristics and network depth: the dataset of 11,500 images across 45 classes is moderate for very deep networks, which may be prone to overfitting when fine-grained inter-class differences are subtle. The applied data augmentation helped alleviate this issue, but future work with larger and more diverse datasets could better unlock the potential of very deep architectures. This indicates that network depth does not guarantee better performance; model complexity must match dataset scale, category differences, and task difficulty. For small-sample, fine-grained plant leaf recognition with minimal category differences, moderately deep networks are often superior to ultra-deep ones. The five-fold cross-validation on EfficientNet-B3 produced a mean accuracy of 99.40% (±0.12%) under the image-level split, consistent with the 99.19% accuracy from the single 70/15/15 split. This consistency suggests that the model’s performance is stable across different random partitions of the dataset at the image level. However, because the cross-validation was also performed at the image level, it does not address the fundamental concern that images of the same leaf or the same plant may appear in both training and test folds. Therefore, these results confirm stability under the current split strategy but do not provide a realistic estimate of plant-level generalization. Plant-level cross-validation remains a critical direction for future work.

### 5.3. Sources of Misclassification

Confusion matrices revealed that misclassifications mainly occurred among morphologically similar cultivars, such as *Ilex crenata* ‘Helleri’, and among hybrids like *I.* × *meserveae* cultivars. These non-random errors reflect intrinsic morphological overlap, highlighting the challenge of distinguishing closely related taxa even with high-performing CNNs. Specifically, high misclassification between *I. crenata* ‘Helleri’ and its relatives stems from analogous leaf traits: both have crenate margins and obtuse apices, with minimal leaf shape/size differences that CNNs struggle to capture. Additionally, small sample sizes of some *I.* × *meserveae* cultivars exacerbate this issue—insufficient representation limits the model’s learning of species-specific traits, leading to misclassification with closely related *Ilex* taxa sharing similar leaf texture and venation. This underscores that CNN performance in plant cultivar classification depends not only on architecture but also on taxon morphological distinctiveness and sample sufficiency, particularly for closely related cultivars/hybrids with subtle differences.

Among the six CNN models, GoogLeNet achieved a macro F1-score of 99.59%, comparable to ResNet50 and only slightly lower than DenseNet variants. DenseNet-169 achieved the highest F1-score (99.65%), demonstrating superior robustness and feature representation, while EfficientNet-B3 obtained a slightly lower F1-score of 99.19%. Morphologically similar taxa, notably *I. crenata* var. *convexa*, *I. latifolia*, and *I. crenata* ‘Helleri’, showed lower F1-scores, especially in some models, primarily due to high interspecific similarity in key leaf morphological traits critical for classification.

### 5.4. Feature Attention and Interpretability

Grad-CAM visualizations confirmed that models focused on biologically meaningful leaf regions, including contours, apex, margins, and venation, while ignoring the background. ResNet, DenseNet, and EfficientNet generated more continuous, comprehensive attention maps than GoogLeNet, reflecting better capture of complete leaf structures, an advantage derived from their architectures, particularly residual and dense connections that shape attention via enhanced feature processing. ResNet’s residual connections mitigate vanishing gradients and preserve multi-scale features, retaining fine details and global structure to guide attention to local discriminative regions and full leaf morphology. DenseNet’s dense connections promote feature reuse by concatenating preceding feature maps, enabling integration of complementary traits and focusing attention on biologically critical overlapping regions. EfficientNet uses compound scaling to balance feature depth and width, refining attention to key leaf traits. Attention patterns adapted to leaf traits: spiny-margined leaves emphasized margins and apex, while elongated leaves focused on the mid-lamina. These observations support the high F1-scores and show that residual and dense connections enhance discriminative capability by optimizing attention to biologically meaningful leaf features.

### 5.5. Limitations and Future Work

Several limitations of this study and corresponding future directions are as follows. First, leaf images were collected from only two sites in Nanjing from cultivated or semi-wild populations, limiting geographic generalizability; future work will collect multi-site data from diverse environments. Second, the image-level split (not by individual plant) may cause optimistic performance estimates due to potential overlap of the same plant across training and test sets; plant-level cross-validation will be adopted. Third, no voucher specimens were collected due to conservation restrictions; herbarium collaboration will be sought to obtain vouchers for critical taxa. Fourth, only 45 of over 600 *Ilex* species were included; the dataset will be expanded to include more species and hierarchical classification will be explored. Fifth, models were evaluated only on clean, preprocessed images; raw field photographs (with clutter, variable illumination, occlusion, and seasonal changes) will be tested and domain adaptation applied. Additionally, attention mechanisms and lightweight networks for mobile applications, multi-organ features (leaves, flowers, fruits), and further dataset diversification will be explored to strengthen practical value for automated plant identification and conservation.

## 6. Conclusions

This study developed a deep learning framework for classifying *Ilex* leaf images using six CNN models: GoogLeNet, ResNet50, ResNet101, DenseNet121, DenseNet169, and EfficientNet-B3. After image preprocessing and Grad-CAM visualization, all models achieved over 99% overall accuracy and near-1.0 F1-scores across 45 *Ilex* taxa under an image-level data split that may overestimate performance because images of the same leaf or same plant could appear in both training and test sets. Therefore, plant-level cross-validation is necessary to obtain a realistic estimate of model generalizability for practical applications. Nevertheless, the results confirm that modern CNN architectures can capture fine-grained leaf morphological features effectively. Grad-CAM verified that models focused on key morphological traits such as veins, shape, and margins, adapting to spiny or elongated leaves. The framework provides an efficient, labor-saving alternative to manual identification for preliminary screening under controlled imaging conditions. The current models were evaluated only on clean, preprocessed images; their performance on raw field photographs remains untested, and the current results are limited to image-level splitting. In future work, we will prioritize plant-level data partitioning and cross-validation to assess true field generalizability, expand datasets under more diverse and challenging conditions, integrate lightweight networks for mobile applications, and explore multi-organ classification using leaves, flowers, and fruits.

## Figures and Tables

**Table 1:** Experimental configuration.

Deep Learning Experimental Environment	
Experimental Platform	Featurize (Cloud Service)
GPU	NVIDIA RTX 4090 (24 GB)
CPU	16 cores (AMD EPYC 9354)
RAM	56 GB
Storage	700 GB
OS/Environment	Docker v20.10.10
Programming Language	Python v3.10.12
Deep Learning Framework	PyTorch v2.0.1

**Table 2:** Hyperparameter setting.

Hyperparameter	Value
Epochs	30
Batch Size	16
Optimizer	Adam
Phase I LR (first 5 epochs)	0.0003
Phase II LR (fine-tune)	0.00003
Weight Decay	1 × 10^−4^
Learning Rate Scheduler	ReduceLROnPlateau (factor = 0.5, patience = 3)
Activation Function	ReLU
Loss Function	Cross-Entropy Loss

**Table 3:** Comparison of image classification model performance (precision, recall, and F1 are macro-averaged).

Model	Accuracy (%)	Precision (%)	Recall (%)	F1 (%)	Parameters (M)
GoogLeNet	99.59	99.60	99.59	99.59	6.6
ResNet50	99.59	99.61	99.59	99.59	25.6
ResNet101	99.48	99.49	99.48	99.48	44.5
DenseNet121	99.65	99.66	99.65	99.65	8.0
DenseNet169	99.65	99.66	99.65	99.65	14.2
EfficientNet-B3	99.19	99.23	99.19	99.19	12.0

## Data Availability

The *Ilex* leaf image dataset is available from the corresponding author upon reasonable request due to germplasm conservation restrictions. The source code and training scripts are publicly available at GitHub: https://github.com/chengJ914/Accurate-Identification-of-Ilex-Aquifoliaceae-Taxa-Based-on-Leaf-Morphology (accessed on 24 April 2026). The five-fold cross-validation fold assignments (including folds.csv) are provided as Supplementary Materials.

## Supplementary Materials

The following supporting information can be downloaded at: https://www.mdpi.com/article/10.3390/plants15091365/s1. The following files are available as Supplementary Materials. folds.csv—fold assignment for every image in the five-fold cross-validation. The source code and training scripts are available at the GitHub repository: https://github.com/chengJ914/Accurate-Identification-of-Ilex-Aquifoliaceae-Taxa-Based-on-Leaf-Morphology (accessed on 24 April 2026). Additional dataset information (plants per taxon, leaves per plant) is provided in Table S1.
