# Supplementary material for: Accurate Identification of Ilex (Aquifoliaceae) Taxa Based on Leaf Morphology Using Deep Learning
Source: Plants (Basel). 2026 Apr 29;15(9):1365. doi: 10.3390/plants15091365 (PMC13165308; doi:10.3390/plants15091365)
Supplement: Supplementary file 1 [file plants-15-01365-s001.zip › supplementary materials.pdf]

Table S1. Basic Information of the Dataset.

Taxon ID	Taxon Name	Parents	Plants	Leaves/Plants	Total Number of Images	Number of Training Images	Number of Test Images	Sample experiment front image	Sample experiment reverse image
1	*Ilex metabaptista*		5	23	228	159	35	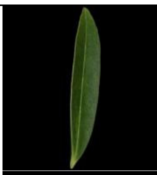	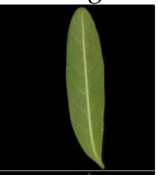
2	'Alaska'	*Ilex aquifolium*	5	30	300	210	45	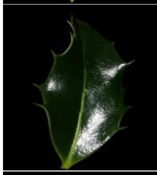	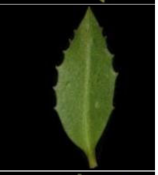
3	'Madame Briot'	*Ilex aquifolium*	5	30	296	207	45	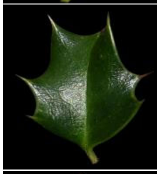	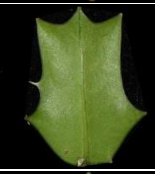
4	'Bacciflava'	*Ilex aquifolium*	4	28	218	152	34	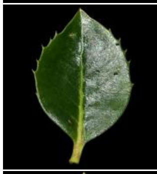	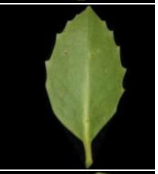
5	'J.C.Van Tol'	*Ilex aquifolium*	4	26	206	144	32	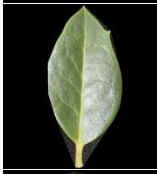	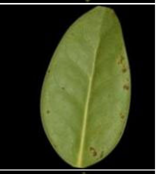
6	*Ilex opaca*		4	23	180	125	28	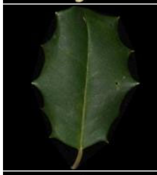	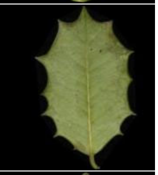
7	'Winter Red'	*Ilex verticillata*	4	27	210	147	32	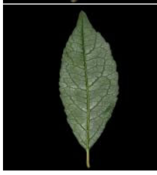	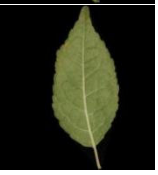
8	'Blue Angel'	（*Ilex aquifolium* × *Ilex rugosa*）× *Ilex aquifolium*）	5	27	268	187	41	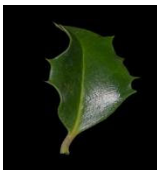	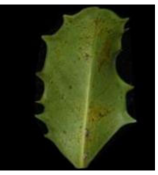
9	'Mesog'	*Ilex cornuta*× *Ilex rugosa*	5	26	260	182	39	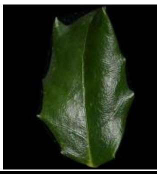	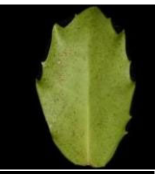

10	*Ilex dimorphophylla*		5	35	348	243	53	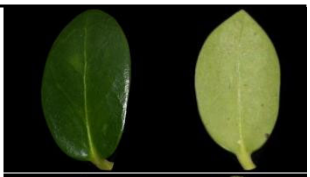
11	'Helleri'	*Ilex crenata*	5	30	296	207	45	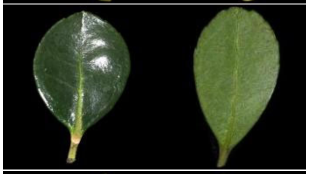
12	*Ilex pernyi*		5	31	302	211	46	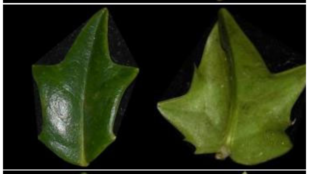
13	'Auburn'	*Ilex latifolia*	2	47	186	130	29	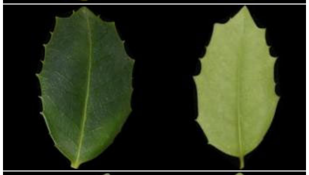
14	*Ilex integra*		5	26	256	179	39	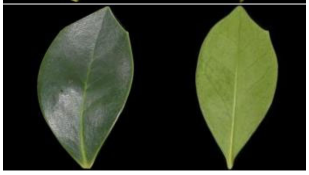
15	'HeckenStar'	*Ilex aquifolium* ′Pyramidalis′ × (*Ilex aquifolium* × *Ilex rugosa*) ′Blue Prince′	2	55	220	154	33	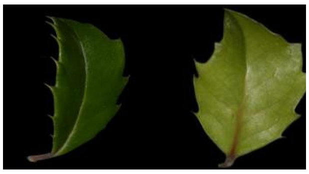
16	'Jersey pinacle'	*Ilex crenata*	5	24	240	168	36	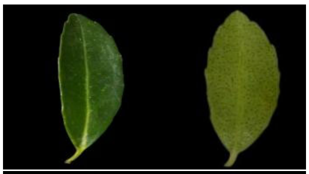
17	'Geisha'	*Ilex crenata*	5	30	300	210	45	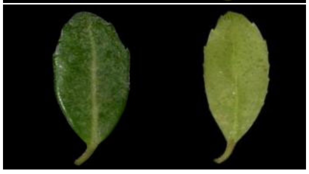
18	'September Gem'	*Ilex ciliospinosa* × (*Ilex aquifolium* × *Ilex pernyi*)	5	21	208	145	32	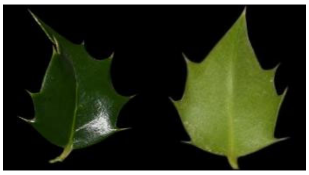
19	'Mesan'	*Ilex aquifolium* × *Ilex rugosa*	4	28	220	154	33	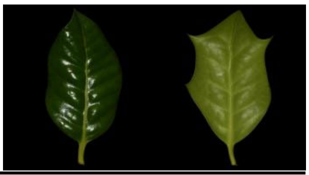

20	*Ilex sanqingshanensis*		5	28	276	193	42	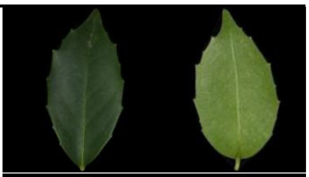
21	*Ilex vomitoria*		5	34	340	237	52	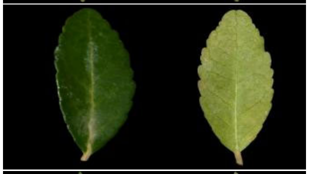
22	*Ilex chinensis Sims*		5	27	264	184	41	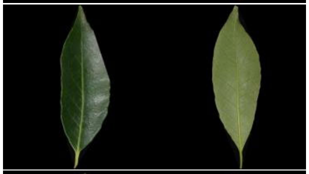
23	'Winter Gold'	*Ilex verticillata*	3	37	218	152	34	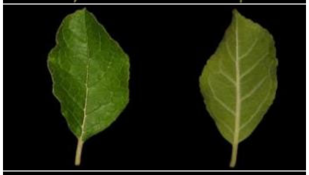
24	*Ilex elmerrilliana*		5	23	226	158	35	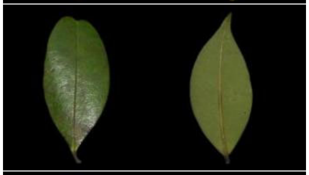
25	*Ilex glabra*		5	24	240	168	36	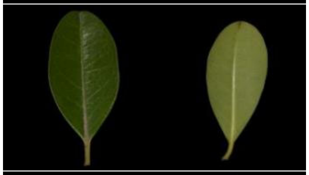
26	*Ilex × dabieshanensis*		4	38	300	210	45	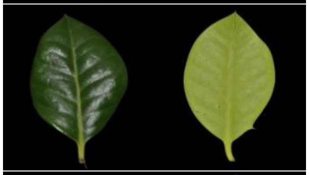
27	*Ilex latifolia*		4	25	198	138	31	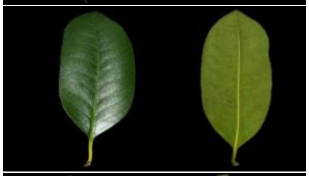
28	*Ilex macrocarpa*		4	35	282	197	43	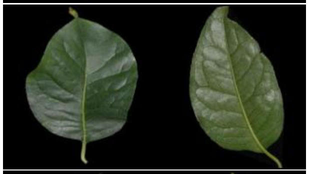
29	*Ilex micrococca*		4	26	206	144	32	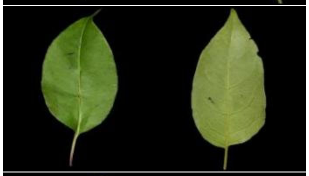
30	*Ilex wilsonii*		5	22	214	149	33	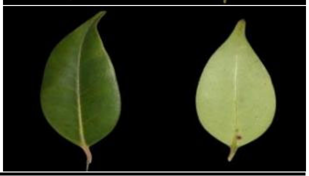

31	*Ilex aquifolium*		4	38	300	210	45	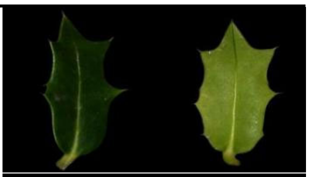
32	'Fortunei'	*Ilex cornuta*	3	50	300	210	45	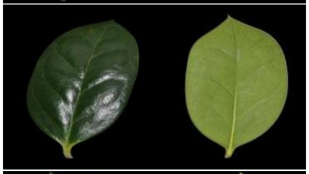
33	*Ilex cornuta*		5	28	278	194	43	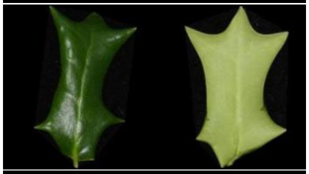
34	*Ilex salicina*		3	34	200	140	30	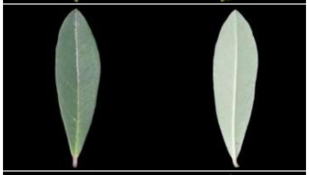
35	*Ilex ficoidea*		5	22	214	149	33	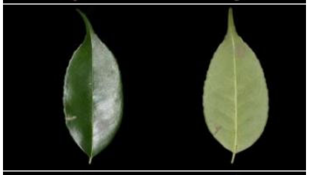
36	*Ilex pubescens*		5	30	300	210	45	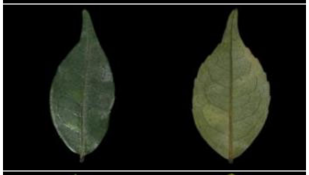
37	'Sky Pencil'	*Ilex crenata*	5	30	300	210	45	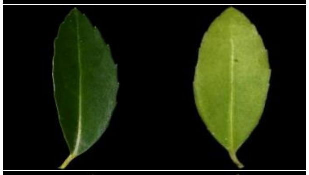
38	'Nellie R. Stevens'	*Ilex cornuta* × *Ilex aquifolium*	4	28	224	156	35	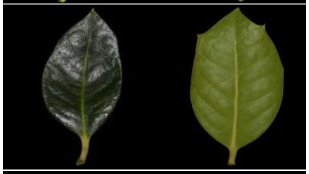
39	*Ilex decidua*		4	33	258	180	40	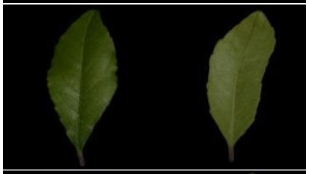
40	'James Swan'	*Ilex cornuta* 'Burfordii' × *Ilex latifolia*	3	35	210	147	32	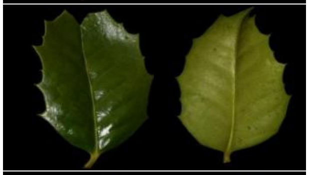
41	′Golden Oakland'	*Ilex* 'Magland'	3	37	220	154	33	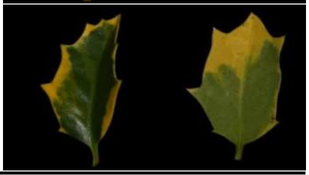

42	*Ilex rotunda*		4	35	274	191	42	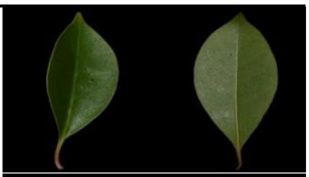
43	*Ilex hanceana*		5	30	300	210	45	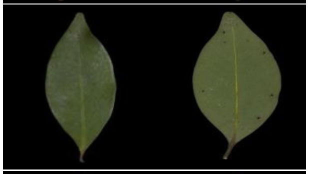
44	'Sunny Foster'	*Ilex* × *attenua ta*	5	32	320	224	48	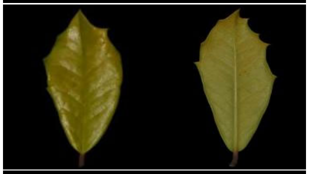
45	*Ilex crenata* var. *convexa* Makino		5	30	296	207	45	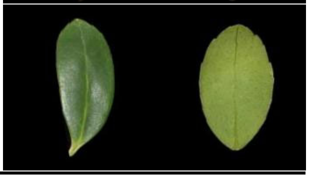
